# Supplementary material for: Role and mechanism of NCAPD3 in promoting malignant behaviors in gastric cancer
Source: Front Pharmacol. 2024 Apr 22;15:1341039. doi: 10.3389/fphar.2024.1341039 (PMC11070777; doi:10.3389/fphar.2024.1341039)
Supplement: Supplementary file 11 [file DataSheet2.ZIP › GSEA/Transcipt factor/my_analysis.Gsea.1599461814734/ALKBH3_TARGET_GENES.html]

Details for gene set ALKBH3\_TARGET\_GENES[GSEA]

|  || Dataset | filtered\_dataset.sample\_info.cls#WT\_versus\_NCAPD3\_MUT |
| Phenotype | sample\_info.cls#WT\_versus\_NCAPD3\_MUT |
| Upregulated in class | WT |
| GeneSet | ALKBH3\_TARGET\_GENES |
| Enrichment Score (ES) | 0.3260859 |
| Normalized Enrichment Score (NES) | 1.6957058 |
| Nominal p-value | 0.020373514 |
| FDR q-value | 0.47371018 |
| FWER p-Value | 1.0 |
Table: GSEA Results Summary

  

Fig 1: Enrichment plot: ALKBH3\_TARGET\_GENES      
 Profile of the Running ES Score & Positions of GeneSet Members on the Rank Ordered List

  

| SYMBOL | TITLE | RANK IN GENE LIST | RANK METRIC SCORE | RUNNING ES | CORE ENRICHMENT || 1 | 64172 | OSGEPL1 | 6 | 1.152 | 0.0545 | Yes |
| 2 | 117145 | THEM4 | 65 | 0.860 | 0.0562 | Yes |
| 3 | 157680 | VPS13B | 78 | 0.838 | 0.0903 | Yes |
| 4 | 50861 | STMN3 | 126 | 0.777 | 0.0958 | Yes |
| 5 | 55900 | ZNF302 | 145 | 0.740 | 0.1205 | Yes |
| 6 | 51018 | RRP15 | 192 | 0.685 | 0.1221 | Yes |
| 7 | 79922 | MRM1 | 209 | 0.669 | 0.1446 | Yes |
| 8 | 5701 | PSMC2 | 244 | 0.636 | 0.1524 | Yes |
| 9 | 26259 | FBXW8 | 318 | 0.583 | 0.1291 | Yes |
| 10 | 51491 | NOP16 | 334 | 0.573 | 0.1474 | Yes |
| 11 | 9617 | MTRF1 | 349 | 0.568 | 0.1663 | Yes |
| 12 | 166785 | MMAA | 369 | 0.553 | 0.1807 | Yes |
| 13 | 57109 | REXO4 | 370 | 0.552 | 0.2090 | Yes |
| 14 | 10426 | TUBGCP3 | 402 | 0.535 | 0.2137 | Yes |
| 15 | 801 | CALM1 | 424 | 0.518 | 0.2249 | Yes |
| 16 | 8607 | RUVBL1 | 451 | 0.506 | 0.2318 | Yes |
| 17 | 6742 | SSBP1 | 501 | 0.472 | 0.2203 | Yes |
| 18 | 1819 | DRG2 | 550 | 0.447 | 0.2082 | Yes |
| 19 | 55245 | UQCC1 | 580 | 0.434 | 0.2093 | Yes |
| 20 | 93134 | ZNF561 | 590 | 0.431 | 0.2247 | Yes |
| 21 | 55181 | SMG8 | 602 | 0.422 | 0.2383 | Yes |
| 22 | 6787 | NEK4 | 621 | 0.412 | 0.2462 | Yes |
| 23 | 5147 | PDE6D | 624 | 0.410 | 0.2657 | Yes |
| 24 | 79807 | GSTCD | 644 | 0.402 | 0.2725 | Yes |
| 25 | 79269 | DCAF10 | 668 | 0.394 | 0.2758 | Yes |
| 26 | 5134 | PDCD2 | 678 | 0.388 | 0.2891 | Yes |
| 27 | 89910 | UBE3B | 693 | 0.381 | 0.2984 | Yes |
| 28 | 55750 | AGK | 697 | 0.380 | 0.3156 | Yes |
| 29 | 134430 | WDR36 | 733 | 0.359 | 0.3085 | Yes |
| 30 | 23640 | HSPBP1 | 735 | 0.359 | 0.3261 | Yes |
| 31 | 3146 | HMGB1 | 831 | 0.295 | 0.2720 | No |
| 32 | 6520 | SLC3A2 | 878 | -0.290 | 0.2534 | No |
| 33 | 3716 | JAK1 | 939 | -0.363 | 0.2282 | No |
| 34 | 8881 | CDC16 | 963 | -0.383 | 0.2311 | No |
| 35 | 6430 | SRSF5 | 1183 | -0.553 | 0.0999 | No |
| 36 | 22931 | RAB18 | 1231 | -0.608 | 0.0968 | No |
| 37 | 5187 | PER1 | 1254 | -0.641 | 0.1135 | No |
Table: GSEA details [plain text format]

  

Fig 2: ALKBH3\_TARGET\_GENES      
 Blue-Pink O' Gram in the Space of the Analyzed GeneSet

  

Fig 3: ALKBH3\_TARGET\_GENES: Random ES distribution      
 Gene set null distribution of ES for **ALKBH3\_TARGET\_GENES**
